# Chemotherapy versus radiotherapy for FIGO stages IB1 and IIA1 cervical carcinoma patients with postoperative isolated deep stromal invasion: a retrospective study

**DOI:** 10.1186/s12885-016-2447-2

**Published:** 2016-07-07

**Authors:** Lei Li, XiaoYan Song, RuoNan Liu, Nan Li, Ye Zhang, Yan Cheng, HongTu Chao, LiYing Wang

**Affiliations:** Department of Gynecologic Oncology, the Affiliated Cancer Hospital of Zhengzhou University, Zhengzhou, 450008 China; Department of Clinical Laboratory, the Third Affiliated Hospital of Zhengzhou University, Zhengzhou, 450052 China

**Keywords:** Cervical cancer, Deep stromal invasion (DSI), Chemotherapy, Radiotherapy

## Abstract

**Background:**

The adjuvant treatment for patients with isolated stromal invasion after radical hysterectomy and pelvic lymph node dissection (PLND) in FIGO stage IB1 and IIA1 cervical carcinoma has not been established. This study assessed the survival outcomes and recurrent patterns in this particular group of patients treated with chemotherapy or radiation-based adjuvant therapy.

**Methods:**

The records 133 IB1 and IIA1 postoperative cervical carcinoma patients with histopathology-confirmed isolated deep stromal invasion (DSI) without any other unfavorable pathological finding between June 2010 and March 2013 were analyzed. Sixty-five patients received postoperative adjuvant four to six cycles of cisplatin-based chemotherapy (CT group) and Sixty-eight received postoperative received postoperative adjuvant radiotherapy (RT group). Treatment-related toxicities were evaluated and disease-free survival (DFS) and overall survival (OS) were analyzed using Kaplan-Meier estimates and statistical significance was determined using the log-rank test.

**Results:**

With a median follow-up of 33.7 months (range 10–62 months), RT group had a significantly improved in DFS rate (*P* = 0.044), but there was no significant difference in overall survival (*P* = 0.437). Upon further analysis, patients with outer 1/3 to full-thickness invasion in chemotherapy group exhibited significantly higher recurrence rates compared to the radiotherapy group. Leukocytopenia, nausea and vomiting were the most frequent short-term complications of chemotherapy, whereas colitis/proctitis and cystitis were more frequent in the radiotherapy group (*P* = 0.000 respectively). No significant differences were found regards to other acute toxicities, including hemoglobin, platelets and ALT/AST, colitis/proctitis, cystitis and dermatitis (*P* = 0.000 respectively). Fewer late severe side effects in the chemotherapy group were observed compared with the radiation group and significant differences were found at colitis/proctitis, cystitis and dermatitis (*P* = 0.000 respectively).

**Conclusion:**

Compared to chemotherapy alone, postoperative RT to FIGO stages IB1 and IIA1 cervical carcinoma patients with isolated DSI can reduce risk of recurrence and with acceptable morbidity.

## Background

Cervical cancer remains one of the most common cancers in women worldwide and more than 85 % of the cancer burden occur in developing countries, around a quarter of the new cases in the world are diagnosed in China every year. The management paradigms for cervical cancer are well established with preference of surgery in early-stage disease patients [1]. After surgery, there are some pathological findings that were regarded as being at high risk of recurrence and warrant necessary postoperative treatment, which including positive pelvic nodes, surgical margin and/or positive parametrium [2–4]. Moreover, if patients with a tumor that is confined to the cervix that display combination of intermediate-risk factors such as a large tumor, lymphovascular space involvement (LVSI), or deep stromal invasion (DSI), are considered to be at risk of recurrence and also need postoperative pelvic radiotherapy (RT).

One frequently encountered situation at clinical practice, however, was that only one of these intermediate risk pathology factors, such as isolated deep stromal invasion without LVSI+ and larger tumor diameter was presented. For this situation, radiotherapy as an adjuvant treatment after radical hysterectomy has been widely used. Moon et al. evaluated the potential benefit of postoperative radiotherapy in women with full-thickness cervical stromal invasion (FTSI) without any other unfavorable pathological finding and found that postoperative radiotherapy could improve pelvic control with acceptable morbidity [5]. As an alternative, the utility of chemotherapy (CT) has also been suggested for postoperative adjuvant therapy. For example Takeshima reported that at their institute, CT alone has been used as postoperative adjuvant therapy for cervical cancer and treatment results suggest the potential role of adjuvant chemotherapy alone for patients with intermediate- and high-risk cervical cancer [6].

Although it has been suggested that adjuvant CT combined with radical hysterectomy and systematic lymphadenectomy has a survival benefit [7] and patients after operation often received adjuvant chemotherapy or radiotherapy in deferent institution, the role of adjuvant treatment has not been extensively investigated. Thus, the purpose of the present study was to evaluate the survival outcomes and in patients with isolated deep stromal invasion treated with chemotherapy and radiotherapy after radical hysterectomy and PLND in stages IB1 and IIA1 cervical carcinoma.

## Methods

### Patients and procedures

The Institutional Review Board of the Cancer Hospital of Zhengzhou University reviewed and approved this study ((approval No.15CT079) and medical records were obtained with informed consent of all patients. The inclusion criteria were: (1) age 35–75 years old; (2) with definite histological diagnosis; (3) normal liver and renal function; (4) acceptable cardiovascular pulmonary and other major organ functions. Exclusion criteria including: (1) age < 36 or > 75 years; (2) any lung, liver, or cardiovascular pulmonary and other major organ dysfunctions; (3) with other high risk factors include parametrial extension, positive pelvic nodes and margins (4) combined these intermittent risk pathology factors; (5) pathologically proven distant metastasis; (6) with any component of neuroendocrine or clear cell differentiation within the tumor wit and another coexisting malignancy. A total of 1577 FIGO stage IB1 and IIA1 cervical cancer patients treated by radical abdominal hysterectomy and lymphadenectomy with/without bilateral salpingo-oophorectomy between June 2010 and March 2013 was performed. 1414 patients with positive pelvic LN or combined with other pathologic risk factor were excluded.143 patients met the including criteria were identified and ten patients without complete follow-up data were excluded. Of the included 133 patients, 65 patients received postoperative chemotherapy (chemotherapy group) and 68 patients received postoperative pelvic radiotherapy (RT group) and concurrent weekly cisplatin as sensitizer.

### Chemotherapy

Sixty-five patients received platinum-based chemotherapy. The paclitaxel plus cisplatin regimen was given every 3 weeks, which consisted of paclitaxel 135 mg/m^2^ over 3 h IV on day 1 and cisplatin 60 mg/m^2^, 2-h i.v. infusion for 4–6 cycles. Toxicity was graded according to the National Cancer Institute common toxicity criteria. Granulocyte colony stimulating factor (GCS-F) was subcutaneously administered at the dose of 5 μg/kg daily in case of WBC less than 4000/μl until recovery. Red blood cell transfusion was administered in case of hemoglobin level below 7 gr/dl.

### External beam RT

Sixty-eight patients after 4 weeks of radical surgery received whole pelvis irradiation with 3D conformal radiotherapy (3D-CRT). The median radiation dose of 50 Gy was delivered in 1.8 to 2.0 Gy fractions once daily for 5 days per week. The pelvic treatment fields generally extended superiorly to include L5. When lateral fields were used, the posterior border encompassed S2. CTV (clinical target volume) was defined as an area of potential microscopic disease and included supravaginal portion, paracervical tissue, common iliac lymph nodes, internal and external iliac lymph nodes, obturator lymph nodes and sacral lymph nodes. Patients in RT groups also received platinum-based concurrent chemotherapy, which consisted of weekly 40 mg/m^2^ intravenous cisplatin.

### Follow-up evaluation

Patients were evaluated every three months for the two year, every six months during the following three years, and annually thereafter. At each visit, bimanual examination and physical examination, and vaginal cytology were performed for the detection of lower genital tract neoplasia. Scans of the abdomen and pelvic region were conducted by ultrasound or CT scan. Suspected cases of recurrent disease were confirmed by biopsy whenever possible. Disease-free survival (DFS) and Overall survival (OS) was calculated from the date of diagnosis until the date of occurrence of disease progression and to the date of death or, for surviving patients, to the date of last follow-up. The cause of deaths due to disease, directly or indirectly from treatment-related complications, and unknown causes was confirmed by correspondence, telephone or medical record review. Surviving patients were censored on the date of last follow-up.

### Statistical analysis

Clinical characteristics of patients, local control, survival, toxicities, and the dosimetric parameter were compared using Fisher exact test for frequencies and the Mann–Whitney *U* test for continuous variables between the two groups. The probabilities of DFS, OS were calculated using the Kaplan-Meier method. Differences between groups were analyzed using the Log-rank statistic. Statistical significance was defined at a level of *P* < 0.05. All analyses were performed using SPSS version 17.0 (SPSS Inc., Chicago, IL).

## Results

### Patient and tumor characteristics

The median age of all patients was 49.0 years (range 36–73) in chemotherapy group and 50.8 years (range 37–75) in RT group. Comparison of between these two groups revealed no statistically significant differences in terms of age (*P* = 0.141), FIGO stage (*P* = 0.474), histology subtype (*P* = 0.525), grade (*P* = 0.556), depth of stromal invasion (*P* = 0.698) and the number of retrieved lymph nodes (*P* = 0.303). The patient characteristics are summarized in Table 1.

**Table 1:** Clinical and pathologic characteristics at chemotherapy group and radiotherapy group

Characteristics	CT (*n = 65*)	RT (*n = 68*)	*P* value
Age (years)	49.0(36–73)	50.8(37–75)	0.141
BMI FIGO stage	25.5 ± 3.7	26.1 ± 3.8	0.754 0.474
IB1 IIA1	22(33.8 %) 43(66.2 %)	28(41.2 %) 40(58.8 %)	
Depth of stromal invasion			0.698
<1/3 1/3-2/3 2/3-full-thickness	23(35.4 %) 26(40.0 %) 16(24.6 %)	21(30.9 %) 30(44.1 %) 17(25.0 %)	
Histology			0.525
Squamous cell carcinoma	59(90.8 %)	63(92.6 %)	
Adenocarcinoma	6(9.2 %)	5(7.4 %)	
Grade			0.556
1	22 (33.8 %)	20 (29.4 %)	
2	27(41.5 %)	29(42. 6 %)	
3	16(24.6 %)	19(27.9 %)	
Number of retrieved lymph nodes	22.8 ± 4.7	23.6 ± 3.9	0.303

### Complications

Acute toxicities, measured from the initiation of treatment to 90 days after completion, were graded according to the National Cancer Institute Common Toxicity Criteria for Adverse Events, version 3.0. Adverse events 90 days after the completion of treatment were graded according to the Radiation Therapy Oncology Group (RTOG) late toxicity scale. Leukocytopenia, nausea and vomiting were the most frequent short-term complications at chemotherapy group (*P* = 0.003 and 0.020), whereas colitis/proctitis and cystitis complications were more frequent in the radiotherapy group (both *P* = 0.000). Platelets, hemoglobin and ALT/AST were common in both group, and no statistically significant difference was found (*P* = 0.198 and 0.151 and 0.776 respectively). The acute toxicities are summarized in Table 2.

**Table 2:** Incidences of acute toxicities at chemotherapy and radiotherapy group according to National Cancer Institute Common Toxicity Criteria for Adverse Events, version 3.0

Grade	CT (*n = 65*)	RT (*n = 68*)	*P* value
Leukocytes			0.003
1	6(9.2 %)	15(22.1 %)	
2	2436.9 %)	34(50.0 %)	
3	32(49.2 %)	15(22.1 %)	
4	3(4.6 %)	4(5.9 %)	
Hemoglobin			0.198
1	30(46.2 %)	39(57.4	
2	31 (47.7 %)	26(38.2 %)	
3	4(6.2 %)	3(4.4 %)	
4	0(0 %)	0(0 %)	
Platelets			0.151
1	38(58.5 %)	47(69.1 %)	
2	20(30.8 %)	18(26.5 %)	
3	6(9.2 %)	3(4.4 %)	
4	1(1.5 %)	0 (0 %)	
ALT/AST			0.776
1	7(10.8 %)	3(4.4 %)	
2	1(1.5 %)	0(0 %)	
3	0(0 %)	0(0 %)	
4	0(0 %)	0(0 %)	
Nausea/vomiting			0.020
1	27(41.5 %)	41(60.3 %)	
2	24(36.9 %)	20(29.4 %)	
3	14(21.5 %)	7(10.3 %)	
4	0(0 %)	0(0 %)	
Colitis/Proctitis			0.000
1	64(100 %)	40(58.8 %)	
2	1(1.5 %)	25(36.8 %)	
3	0(0 %)	3(4.4 %)	
4	0(0 %)	0(0 %)	
Cystitis			0.000
1	65(100 %)	49(70.1 %)	
2	0(0 %)	15(22.1 %)	
3	0(0 %)	4(5.9 %)	
4	0(0 %)	0(0 %)	

As for the incidence of late toxicities in chemotherapy and radiation therapy group according to Radiation Therapy Oncology Group-European Organization for Research and Treatment of Cancer (RTOG/EORTC) criteria. Fewer late severe side effects in the chemotherapy group were observed compared with the radiation group and significant differences were found at colitis/proctitis, cystitis and dermatitis (*P* = 0.000, 0.000 and 0.000, respectively), but no patients experienced grade 3/4 skin reaction and treatment-related fistula formation and small-or large-bowel obstruction were recorded in the RT group as shown in Table 3.

**Table 3:** Incidences of late toxicities in chemotherapy and radiotherapy group according to RTOG/EORTC criteria

Grade	CT (*n = 65*)	RT (*n = 68*)	*P* value
Colitis/Proctitis			0.000
0	65(100 %)	34(44.1 %)	
1	0(0 %)	23(33.8 %)	
2	0(0 %)	10(20.6 %)	
3	0(0 %)	1(1.5 %)	
4	0(0 %)	0(0 %)	
Cystitis			0.000
0	65(100 %)	33(48.5 %)	
1	0 (0 %)	25(22.1 %)	
2	0(0 %)	9(11.8 %)	
3	0(0 %)	0(0 %)	
4	0(%)	0(0 %)	
Dermatitis			0.00
0	65(100 %)	38(55.9 %)	
1	0(0 %)	22(32.4 %)	
2	0(0 %)	8(11.8 %)	
3	0(0 %)	0(0 %)	
4	0(0 %)	0(0 %)	

### Clinical outcomes and impact of treatment on survival

The median follow-up for patients was 33.7 months (range 10–62 months). All time intervals were measured from the time of diagnosis. In the chemotherapy group, 59 of 65 patients were alive without evidence of disease and one patients died from cervical cancer and five (7.7 %) patients developed a recurrence after a median time of 30.8 months (range, 10–58 months) and received brachytherapy, pelvic or paraaortic RT. Of these five patients with recurrence, three patients had vaginal recurrences; one patient with peripheral pelvic wall recurrence and one patient had distant failure and died. In RT group, after a median time of 36.4 months (range, 14–62 months), one (1.5 %) out of 68 patients relapsed in the lung was observed and died.

**Fig. 1:**
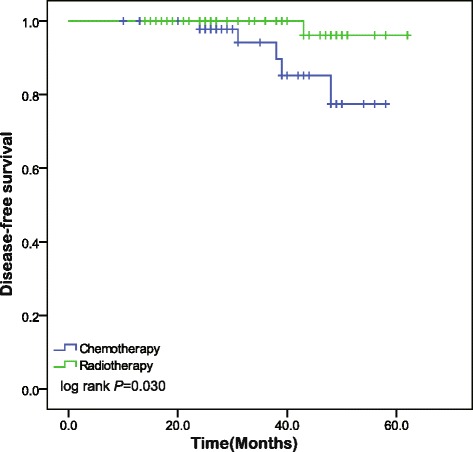
Kaplan-Meier survival curves of disease free survival (DFS) at chemotherapy and radiotherapy group

**Fig. 2:**
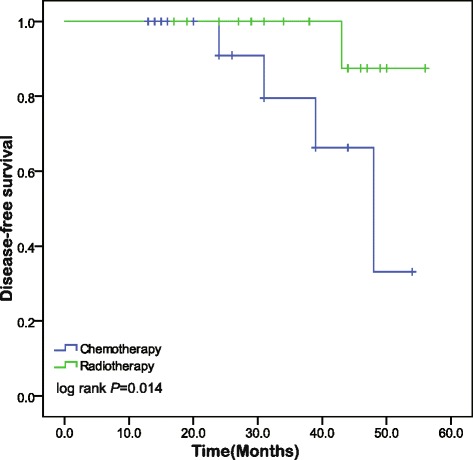
Kaplan-Meier survival curves of disease free survival (DFS) stratified by depth of stromal invasion. It was the subgroup of patients with stromal invasion depth at 2/3 to full-thickness that made significant difference at these groups

Statistically significant difference was observed when different adjuvant treatment was compared. DFS at chemotherapy group and the radiotherapy group were 77.4 % and 96.2 % according to Kaplan-Meier analysis and DFS rates in radiotherapy group were significantly increased compared to those in the chemotherapy group (*P* = 0.030) (Fig. 1). In order to find underlying variable that makes the difference, we stratified the analysis according to the stromal invasion depth and it revealed more details. It is the subgroup of patients with invasion greater outer to full-thick invasion among radiotherapy group were significantly increased compared to those in the chemotherapy groups (*P* = 0.014) (Fig. 2). This correlation between status of invasion depth and recurrence was enhanced when we found that the recurrence occurred almost in patients with full-thick invasion. The five-year OS rate for the patients treated with CT and RT group were 95.5 % and 95.7 %, respectively, and no statistically significant difference was found (*P* = 0.783).

## Discussion

The goal of postoperative radiotherapy (PORT) in patients with a combination of minor risk factors according to the initial report of Gynecologic Oncology Group (GOG) randomized trial was to reduce the risk of recurrence and improve survival in patients. But, due to the limitedness of access to radiotherapy and the on the discretion of the gynecologic oncologist [8], cervical cancer patients with postoperative intermediate pathologic risk factor in many countries and clinical centers often be treated with chemotherapy after radical hysterectomy [9, 10] and some data support the clinical usefulness of chemotherapy as a primary treatment for cervical cancer. For example, in Takeshima study, three courses of bleomycin, vincristine, mitomycin, and cisplatin were administered adjuvantly to 30 patients with intermediate-risk (stromal invasion more than 50 %) and five courses for high-risk cases. Their treatment results suggest the potential role of adjuvant chemotherapy alone for patients with cervical cancer [6]. Hosaka M et al. also found that in patients with intermediate risks chemotherapy has the equivalent therapeutic effects as RT but with fewer postoperative complications [11].

However, these reports all evaluated the patients with combination of intermediate risks. In fact, patients without a combination of intermediate risks, but only one of the intermediate risks were often encountered in clinical practice. DSI, for example, was the often encountered pathology situation. In a retrospective study, Shimada et al. reported that all 21 of their patients with isolated DSI after radical hysterectomy and PLND were not administered PORT and experienced no recurrence [12]. They suggested that isolated DSI should not be used to select patients for adjuvant radiation and that DSI could be excluded as a prognostic factor. But more study found that the relative risk of recurrence was proportional to the depth of stromal invasion, and 5-year DFS in patients with an isolated outer one-third invasion was reported to be about 80 % to 90 % in a GOG study [13, 14]. However, few studies have evaluated the outcomes of different adjuvant therapy strategies in this particular group of patients and it is not certain whether chemotherapy alone improves the survival and standard postoperative adjuvant therapy is needed.

In this study we compared the DFS of patients with deep stromal invasion and received postoperative adjuvant chemotherapy with patients who received postoperative adjuvant radiation. Contrary to Lee findings that adjuvant chemotherapy in patients with FIGO stage IB1 and IIA1 cervical cancer and surgically confirmed intermediate risk factors may be effective [7], the Kaplan-Meier survival analysis in our study demonstrated that the postoperative recurrence rate was significant reduced in the chemoradiation group. The DFS were higher in patients treated with postoperative adjuvant radiation than in those treated with adjuvant chemotherapy and differed significantly with survival prolonged DFS 96.2 % (*P* = 0.030), indicating that the reduction in recurrence will certainly benefit a number of patients with this pathologic factor and patients therefore might benefit from pelvic irradiation.

Upon further stratification based on invasion depth, the data showed that patients did demonstrate inferior responses to chemotherapy compared to chemo radiotherapy and we found that the recurrence occurred almost in patients with full-thick invasion, which indicated that women with full-thick were more likely to develop recurrence. Although these subsets were small, the present results suggest that full-thick invasion alone might be a definitive indication for postoperative radiotherapy and patients with full-thick might be allocated to the postoperative radiotherapy. Postoperative adjuvant therapy for this subgroup of patients cannot replaced by chemotherapy alone and the adjuvant therapy for IB1and IIA1 patients, even not combined with other intermediate risks, should be tailored according to stromal deepness status. However, improved DFS at radiotherapy group observed in our finding may also contributed to the combined treatment method, as the radiation group was given concurrent weekly cisplatin as a radiation sensitizer, the benefit of concurrent chemoradiation over chemotherapy will remains unclear [15].

It cannot deny the fact that the risk of complications was higher in patients administered to RT group. A corresponding incidence rate of late toxicities was observed in RT group than in CT group, which was consistency to previous report study [3]. But no serious toxicity requiring surgical intervention for intestinal obstruction or formation of a fistula, and the frequencies of late toxicities developed in the RT group in the present study are more likely to result in early and late complications, such as gastrointestinal and genitourinary complications, while only mild to moderate bone marrow depression in chemotherapy group were significantly observed. However, as the rate of severe complications have been reported divergent retrospective studies, in stage IB–IIA cervical carcinoma with acceptable morbidity in many surgery plus PORT. As a proportion of patients diagnosed at younger ages and early disease stages, postoperative RT may extinguish the advantages of surgical therapy [16, 17]. The risk-to-benefit ratio must always be considered in determining adjuvant treatment modality. In order to reduce the morbidity that may be caused by aggressive multimodality therapy, it seems important to conduct randomized trials to verify the effectiveness of these strategy adjuvant therapies may improve survival but are associated with several adverse effects and toxicities [18, 19].

In conclusion, the results of the present study strongly support the use of radiation in patients. However, because of the non-prospective design of the study, the analysis in this study suffers from unforeseen biases and we cannot draw the final conclusion regarding the respective effects of the two adjuvant treatment modalities on overall survival. A prospective randomized trial of CT versus RT as an optional adjuvant therapy to patients is necessary in future study to confirm this finding. Treatment strategies and different protocols of chemotherapy and pelvic radiation are required further with a prospective study.

## Conclusions

Our study indicated postoperative RT administered to patients with isolated DSI can reduce the risk of locoregional recurrence and improves the disease-free survival, suggesting that adjuvant postoperative treatment should be tailored according to stromal deepness status. The findings of this study are important for further investigations.

## Abbreviations

3D-CRT, 3D conformal radiotherapy; CT, chemotherapy; CTV, clinical target volume; DFS, disease-free survival; DSI, deep stromal invasion; FTSI, full-thickness cervical stromal invasion; GCS-F, granulocyte colony stimulating factor; GOG, Gynecologic Oncology Group; LVSI, lymphovascular space involvement; NCCN, National Comprehensive Cancer Network; OS, overall survival; PLND, pelvic lymph node dissection; PORT, postoperative radiotherapy; RT, radiotherapy; RTOG, Radiation Therapy Oncology Group; RTOG/EORTC, Radiation Therapy Oncology Group-European Organization for Research and Treatment of Cancer
